# Fluid overload in hemodialysis patients: a cross-sectional study to determine its association with cardiac biomarkers and nutritional status

**DOI:** 10.1186/1471-2369-14-266

**Published:** 2013-12-02

**Authors:** Marlies Antlanger, Manfred Hecking, Michael Haidinger, Johannes Werzowa, Johannes J Kovarik, Gernot Paul, Manfred Eigner, Diana Bonderman, Walter H Hörl, Marcus D Säemann

**Affiliations:** 1 Department of Internal Medicine III - Clinical Division of Nephrology and Dialysis, Medical University of Vienna, Währinger Gürtel 18-20, 1090, Vienna Austria; 2 Department of Internal Medicine III, Donauspital KH SMZ Ost, Vienna Austria; 3 Department of Internal Medicine I, Sozialmedizinisches Zentrum Süd - Kaiser-Franz-Josef-Spital mit Gottfried von Preyer’schem Kinderspital, Vienna Austria; 4 Department of Internal Medicine II - Clinical Division of Cardiology, Medical University of Vienna, Vienna Austria

**Keywords:** Hemodialysis, Fuid overload, Cardiovascular, Inflammation, Bioimpedance

## Abstract

**Background:**

Chronic fluid overload is associated with higher mortality in dialysis patients; however, the link with cardiovascular morbidity has not formally been established and may be influenced by subclinical inflammation. We hypothesized that a relationship exists between fluid overload and [i] cardiovascular laboratory parameter as well as between fluid overload and [ii] inflammatory laboratory parameters. In addition, we aimed to confirm whether volume status correlates with nutritional status.

**Methods:**

We recorded baseline characteristics of 244 hemodialysis patients at three hemodialysis facilities in Vienna (*Austria*) and determined associations with volume measurements using the body composition monitor (*Fresenius/Germany*). In one facility comprising 126 patients, we further analyzed cardiovascular, inflammatory and nutritional parameters.

**Results:**

We detected predialysis fluid overload (FO) in 39% of all patients (n = 95) with FO defined as ≥15% of extracellular water (ECW). In this subgroup, the absolute FO was 4.4 +/-1.5 L or 22.9 ± 4.8% of ECW. A sub-analysis of patients from one center showed that FO was negatively associated with body mass index (r = -0.371; p = <0.001), while serum albumin was significantly lower in fluid overloaded patients (p = 0.001). FO was positively associated with D-Dimer (r = 0.316; p = 0.001), troponin T (r = 0.325; p < 0.001), and N-terminal pro-B-type natriuretic peptide (r = 0.436; p < 0.001), but not with investigated inflammatory parameters.

**Conclusions:**

Fluid overload in HD patients was found to be lower in patients with high body mass index, indicating that dry weight was inadequately prescribed and/or difficult to achieve in overweight patients. The association with parameters of cardiovascular compromise and/or damage suggests that fluid overload is a biomarker for cardiovascular risk. Future studies should determine if this applies to patients prior to end-stage renal disease.

## Background

Patients with end-stage renal disease are exposed to extreme volume shifts and thereby cardiovascular strain as a consequence of interdialytic weight gain, fluid removal during hemodialysis and also chronic fluid overload [1,2]. Fluid overload leads to distorted hemodynamic conditions, and most probably higher cardiovascular morbidity [3,4]. Several biomarkers of cardiovascular risk have been studied in hemodialysis patients during the recent years, yet their clinical significance remains vague. Contradicting results regarding pathological cut-off values and prognostic information have been shown [5-8].

To date, only serum N-terminal brain natriuretic peptide (BNP) levels are known to be strongly associated with fluid overload [3,9,10] and have recently even been suggested as a guide for fluid management in hemodialysis patients [11]. Troponin T (TnT) and D-Dimer have been shown to be elevated in the dialysis population in general [12-14], but a direct association with fluid overload has not been demonstrated for TnT [3], or has not yet been studied, as in the case of D-dimer. Thus, further research regarding the association between biomarkers for cardiovascular disease (CVD) and fluid overload is warranted.

Chronic subclinical inflammation may be an additional contributing factor in the intertwined processes of repeated fluid removal and cardiac stress [15,16]. It has been suggested that hemodialysis patients are exposed to high endotoxin levels in the blood, possibly due to repeated bacterial translocation from the gut as a consequence of intradialytic changes in blood pressure and/or tissue perfusion [15]. However, it currently remains speculative whether interdialytic weight gain with high ultrafiltration rates and possibly higher intradialytic blood pressure decline, or chronic fluid overload which is not known to be related to intradialytic hypotension, is more likely to predispose patients to increased inflammation and/or cardiac stress.

Fluid overload has recently been shown to result in adverse outcomes for hemodialysis patients [2,17-19]. For many years, a normohydrated fluid status has been regarded an issue of utmost importance to reduce deranged fluid homeostasis and cardiac morbidity and mortality [17,18,20,21]. Adequate fluid status assessment is a critical element in the accurate prescription of hemodialysis and a substantial amount of research - most notably in the field of readily available bioimpedance devices - has taken place within the past couple of years [22-25]. These methods allow for the important differentiation between chronic fluid overload (the amount of residual postdialysis volume overload) [2,26] and interdialytic weight gain (the amount of fluid gained between the end of the dialysis session and the beginning of the next) [1,19,27].

We have recently assessed fluid status in three hemodialysis centers in Vienna, Austria, using the body composition monitor (BCM), a bioimpedance monitoring tool [23]. All three institutions subsequently participated in the ‘BVM-Reg’ study on dry-weight reduction [28], which investigated whether blood volume monitoring (BVM)-adjusted ultrafiltration rates might reduce intradialytic symptoms associated with a rapid ultrafiltration process in fluid overloaded hemodialysis patients [29,30].

In the present study, we aimed at presenting a cross-sectional overview of fluid status in our multicenter patient cohort, hypothesizing that we might be able to confirm the previously shown association between fluid overload and low body mass [2]. Furthermore, we wanted to confirm our hypothesis that fluid overload might be linked with laboratory markers of cardiovascular and/or inflammatory strain.

## Methods

The study was performed in three maintenance hemodialysis centers in Vienna, Austria, comprising 144, 72 and 72 dialysis patients, respectively. The largest center was the maintenance hemodialysis center of the Medical University of Vienna (MUV), while the 2 smaller centers were linked to teaching hospitals of the MUV. As part of the prospective BVM-Reg study which dealt with blood volume monitoring-regulated dry-weight reduction in fluid overloaded hemodialysis patients [28], all our patients routinely underwent BCM measurements. Approval from two independent local Ethics Committees was obtained prior to study initiation (EK#365/2011 [Medical University of Vienna] and EK11-2221211 [City of Vienna]).

### Patient eligibility

All chronic hemodialysis patients at the participating centers were eligible for the BCM measurement. The department heads of all three dialysis centers previously agreed to establish BCM measurements as part of their routine dialysis practice. Since baseline data were recorded in an anonymous form, written and informed consent was only obligatory for fluid-overloaded hemodialysis patients who subsequently underwent dry weight reduction. Our patients were therefore allowed to decline the BCM measurement, as they can refuse any other routine procedure, but did not have to provide informed consent.

### Bioimpedance monitoring

Patients underwent standardized evaluation of their fluid status with the BCM, a portable bioimpedance monitor (Fresenius Medical Care, Bad Homburg, Germany). All measurements were carried out after the short interdialytic interval. Patients were placed in supine position for about 3–5 minutes before the start of the dialysis session. Electrodes were attached to the hand and foot contralateral to the dialysis fistula or graft, and the measurement was conducted as described in the manufacturer’s manual. Clinical data (patient’s sex, age, predialytic weight, height, ultrafiltration rate) were documented using a standardized form. All measurements were accomplished within a time frame of ± 1 week before or after blood collection (center 1). Pre-dialysis fluid overload can be described as an absolute value (in liters) or as a relative variable reflected by the expansion of the extracellular water (ECW) which is then calculated as Rel FO = FO/ECW × 100%. Prior publications have suggested an expansion ≥15% ECW as defining for Rel FO [2].

### Laboratory data

As part of the quarterly blood sampling policy in the University-associated center, we collected routinely determined baseline laboratory data from 126 patients in that particular center after a long interdialytic interval. We determined blood counts, blood chemistry, coagulation parameters, dialysis quality (Kt/V), renal osteodystrophia parameters (phosphate, calcium, parathyroid hormone, vitamin D levels) and inflammatory and cardiovascular markers (C-reactive protein (CRP), Troponin T (TnT), fibrinogen, D-Dimer and NT-proBNP). C-reactive protein, D-Dimer and serum amyloid A (SAA) were measured with a latex agglutination method (reference values <1 mg/dl [CRP], <0.5 μg/ml [D-Dimer]); (reference range 0–6.4 mg/l [SAA]). TnT was determined by an enhanced chemiluminescence immunoassay (reference range 0.00 - 0.03 ng/ml), and NT-proBNP was measured with an immunologic test (reference range 0–125 pg/ml). All laboratory values were analyzed at the Clinical Institute for Laboratory Medicine at the Medical University of Vienna.

### Outliers

Prior to statistical analyses, patients exceeding percental fluid overload of three standard deviations above or below the mean were excluded (n = 1 patient). Cut-offs for laboratory parameters for which regression analyses were planned, were defined as values exceeding 15 times the upper range of normal. This led to the following exclusions: C-reactive protein (cut-off >15 mg/dl): n = 2 patient, D-Dimer (cut-off >7.5 μg/ml): n = 2 patients, TnT (cut-off >0.45 ng/ml): n = 4 patients, and serum amyloid A (cut-off >96 mg/l): n = 11 patients. As nearly all hemodialysis patients met this criterion regarding NT-proBNP, the definition was abandoned for this parameter. We calculated the median value for NT-proBNP, and compared the resulting groups.

### Statistical analysis

Descriptive statistics were used to report baseline patient characteristics. Results regarding parametric variables are presented as the mean with standard deviation (SD) or median with interquartile ranges (IQR) if values were not normally distributed. Categorical variables are expressed as percentage or ratio.

To assess differences regarding fluid status between the participating centers, one-way ANOVA was used. Subsequently, patients from center 1 were divided into two groups, normohydrated and fluid overloaded patients, with the cut-off set at ≥15% Rel FO. Dehydrated patients with < -10% ECW were thus included into the normohydrated group, as in a prior study [2].

Differences between the fluid overloaded and normohydrated groups were determined by Chi-square test for categorical variables. For numerical variables, Mann–Whitney-U Test was used for non-normally distributed values (C-reactive protein, D-Dimer, troponin T, N–terminal proBNP and serum amyloid A); Student’s t-test was applied if values showed Gaussian distribution.

Analyses of association: Spearman’s rank correlation coefficient was utilized to measure the dependence between fluid overload and non-normally distributed biomarkers. For the association analysis between fluid overload and body mass index, Pearson’s correlation coefficient was applied. Additionally, linear regression modeling was used.

Further, the median was calculated for NT-proBNP and the ensuing two groups (above and below the median) compared with regard to fluid overload.

Results were considered statistically significant at a P-value <0.05. The IBM SPSS System for Windows version 19.0.0 (SPSS, Inc., 2010, Chicago, IL) was used for all analyses.

## Results

### Fluid status in the participating centers

**Figure 1:**
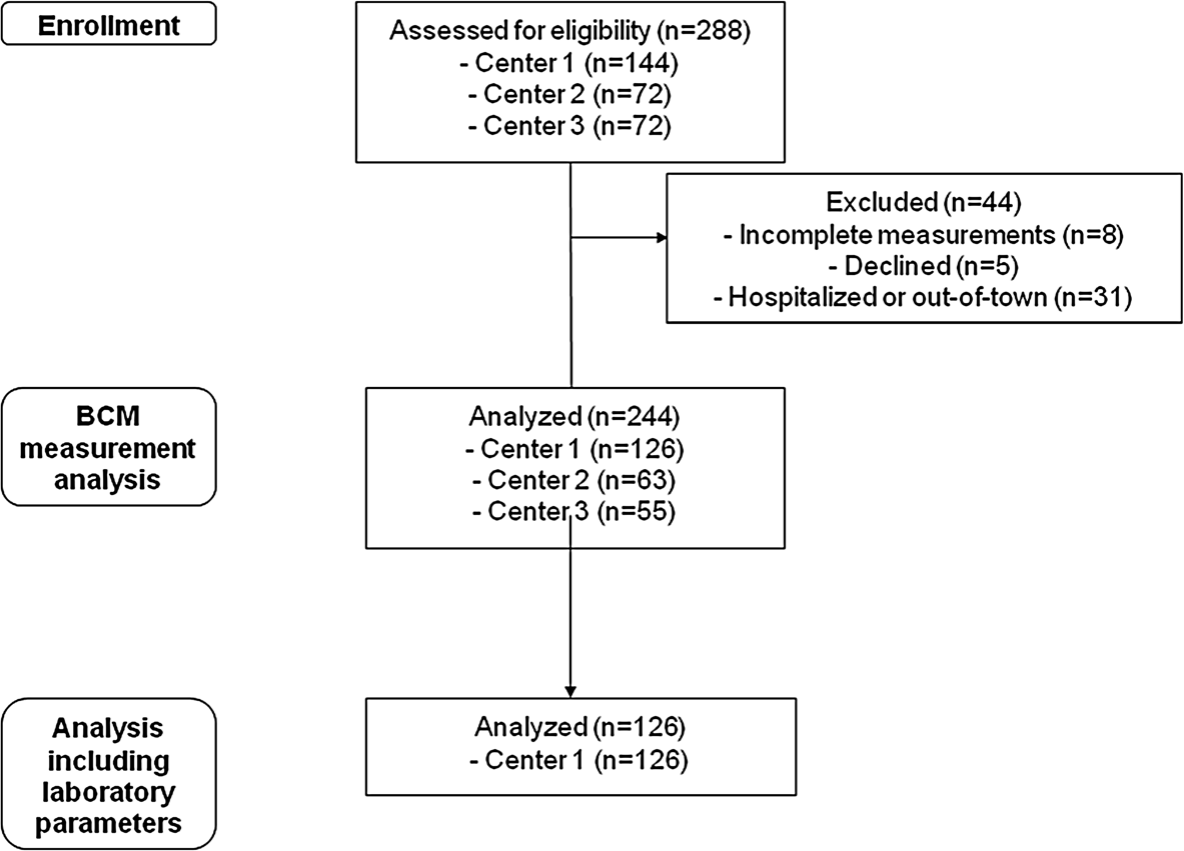
Patient enrollment according to CONSORT flowchart.

288 patients were eligible for bioimpedance measurement, and 252 patients underwent assessment (Figure 1). 36 patients were not measured with the BCM due to various reasons (hospitalized, out-of-town at the time of the measurement, declined). BCM measurements were incomplete in 8 patients, and are not described. Here we report on 126/144 patients in center 1, 63/72 in center 2 and 55/72 in center 3.

As shown in Table 1, 39% of all patients met the definition of pre-HD fluid overload (≥15% Rel FO). Specifically, 43%, 32% and 36% of all measured patients in study centers 1, 2, and 3, respectively, were fluid overloaded. We observed no significant center difference regarding the absolute and relative amount of fluid overload. However, patients from centers 2 and 3 were heavier than patients from center 1, as reflected by dry weight, body mass index and absolute values for ECW.

**Table 1:** Fluid status assessment in participating centers

**Patient characteristic**	**All centers**	**Center 1**	**Center 2**	**Center 3**	**P-value**
	**(n = 244)**	**(n = 126)**	**(n = 63)**	**(n = 55)**	
Dry weight [kg]	75.0 ± 19.2	72.6 ± 17.4	77.2 ± 20.4	80.3 ± 19.2	**0.011**
Body mass index [kg/m^2^]	25.9 ± 5.7	25.0 ± 5.4	26.5 ± 6.1	27.4 ± 5.7	**0.023**
UF [L]	1.8 ± 1.3	1.5 ± 1.0	2.3 ± 1.4	1.9 ± 1.3	**<0.001**
Fluid overloaded pre-HD	39%	43%	32%	36%	0.282
FO pre-HD [L]	2.5 ± 2.2	2.6 ± 2.3	2.6 ± 2.3	2.3 ± 1.8	0.639
FO pre-HD [% ECW]	12.6 ± 10.1	13.1 ± 10.8	12.7 ± 9.9	11.6 ± 8.6	0.556
FO post-HD [% ECW]	2.9 ± 12.3	4.6 ± 12.4	0.9 ± 12.7	1.4 ± 11.5	0.092
ECW pre-HD [L]	18.7 ± 4.5	18.2 ± 4.8	18.6 ± 4.2	20.1 ± 3.7	**0.046**

*Abbreviations*: *UF* ultrafiltration, *FO* fluid overload, *ECW* extracellular water, *HD* hemodialysis.

Percentage for categorical data, mean ± standard deviation for normally distributed numerical variables, median and interquartile ranges for non-normally distributed numerical variables.

As shown in Table 2, absolute values of pre-HD fluid overload in overhydrated patients from center 1 amounted to 4.37 ± 1.52 L, compared to a pre-HD fluid excess of 1.00 ± 1.37 liters in the normohydrated group (p < 0.001).

**Table 2:** Comparison of fluid overloaded and normohydrated patients from center 1

**Patient characteristic**	**Fluid overloaded (n = 56)**	**Normohydrated (n = 70)**	**P-value**
Sex [male]	68%	54%	0.137
Age [years]	61.1 ± 17.3	56.4 ± 17.8	0.110
Vascular access [AVF]	76%	87%	0.102
BMI [kg/m^2^]	23.1 ± 4.4	26.4 ± 5.7	**0.001**
Weight pre-HD [kg]	69.6 ± 16.6	75.5 ± 18.2	0.076
Weight post-HD [kg]	68.3 ± 16.5	73.9 ± 17.9	0.092
UF [L]	1.3 ± 1.0	1.6 ± 1.1	0.144
FO pre-HD [L]	4.4 ± 1.5	1.0 ± 1.4	**<0.001**
FO pre-HD [% ECW]	22.9 ± 4.8	5.8 ± 7.7	**<0.001**
FO post-HD [% ECW]	14.9 ± 7.9	- 3.0 ± 9.1	**<0.001**
ECW pre-HD [L]	18.8 ± 4.0	17.8 ± 5.3	0.290
No. of antihypertensives	3.0 ± 1.7	2.8 ± 1.7	0.700
Systolic BP pre-HD [mmHg]	137.7 ± 23.3	134.6 ± 18.8	0.413
Diastolic BP pre-HD [mmHg]	72.9 ± 15.5	73.5 ± 14.8	0.829
Protein [g/l]	64.9 ± 5.2	66.8 ± 5.3	0.056
Albumin [g/l]	36.4 ± 3.8	38.5 ± 2.9	**0.001**
C-reactive protein [mg/dl]	1.0 (0.3 – 2.3)	0.5 (0.2 – 1.6)	0.129
Hemoglobin [g/dl]	10.2 ± 1.1	10.3 ± 1.2	0.491
Fibrinogen [mg/dl]	389.2 ± 125.6	418.3 ± 129.5	0.221
D-Dimer [μg/ml]	1.2 (0.6 – 2.1)	0.7 (0.3 – 1.7)	**0.021**
Troponin T [ng/ml]	0.07 (0.05 – 0.17)	0.05 (0.03 – 0.08)	**0.020**
NT-proBNP [pg/ml]	10436.5 (4239.3 – 35000)	4485.0 (1956.7 – 11979.5)	**0.001**
Serum amyloid A [mg/l]	14.5 (6.5 – 34.2)	7.6 (5.2 – 25.1)	0.149
Kt/V	1.5 ± 0.4	1.5 ± 0.3	0.596
Charlson comborbidity index	4.96 ± 2.27	4.06 ± 2.04	**0.024**

*Abbreviations*: *yrs* years, *AVF* arteriovenous fistula, *BMI* body mass index, *HD* hemodialysis, *UF* ultrafiltration, *FO* fluid overload, *ECW* extracellular water, *No* number, *BP* blood pressure, *NT-proBNP* N-terminal pro-brain natriuretic peptide, *Kt/V* dialysis adequacy.

Percentage for categorical data, mean ± standard deviation for normally distributed numerical variables, median and interquartile ranges for non-normally distributed numerical variables.

### Association between fluid overload and patient characteristics

When comparing sex and age between normohydrated and fluid overloaded patients, we observed no significant differences (p = 0.137 for sex and p = 0.110 for age, Table 2). Furthermore, when comparing short interdialytic intervals, there was no significant difference in the interdialytic weight gain (IDWG) between the fluid overloaded and normohydrated groups, represented by ultrafiltration volume on the day of the BCM measurement (1.31 ± 0.99 L vs. 1.59 ± 1.08 L, p = 0.144).

**Figure 2:**
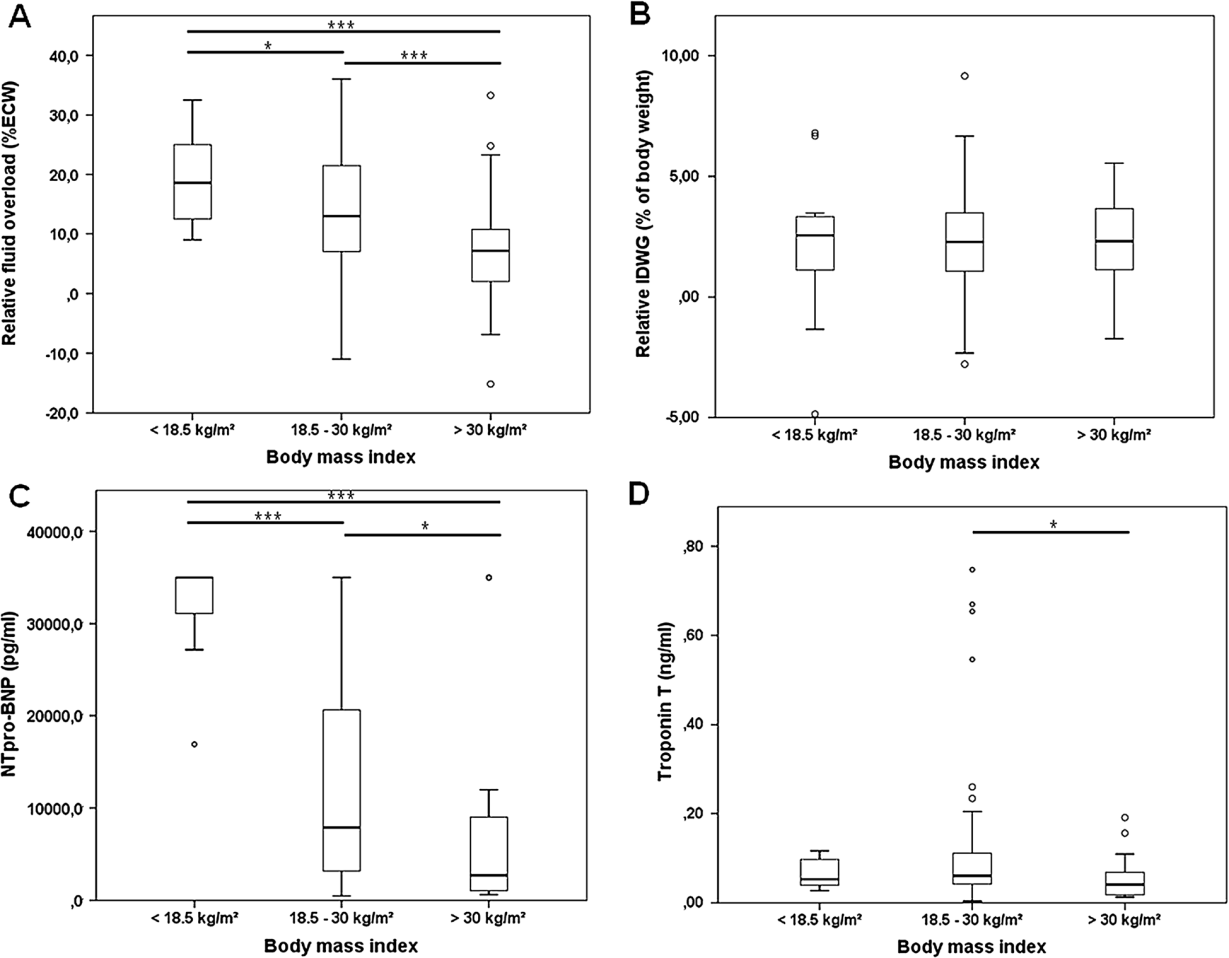
**Patient groups were compared according to BMI (<18.5 kg/m**^**2**^**, n = 14; 18.5-29.9 kg/m**^**2**^**, n = 173; ≥30 kg/m**^**2**^**, n = 56). (A)** Student’s t-Test for **(A)** relative fluid overload and **(B)** relative interdialytic weight gain. Kruskal-Wallis test was used for **(C)** NT-proBNP and **(D)** troponin T.

A negative association was detected for body mass index and fluid overload (Figure 2). A linear regression model showed this direct inverse relationship to be significant (r = -0.371/p < 0.001, Additional file 1: Figure S1) and the patient group with a BMI >30 kg/m^2^ had highly significant lower relative fluid overload values, while no difference was observed for relative interdialytic weight gain (Figure 2). Serum albumin levels were significantly lower in the fluid overloaded patient collective. Similarly, patient comorbidities represented by the Charlson comorbidity index (CCI) [31], showed a significant association with fluid overload, as the CCI was significantly lower in patients exhibiting normohydration (mean 4.06 ± 2.04) compared to those with percental fluid overload (mean 4.96 ± 2.27 (p = 0.024)).

Lower body mass index was further associated with higher NT-proBNP levels (Figure 2). A multiple regression analysis was run to predict NT-proBNP from percental fluid overload and comorbidities represented by the Charlson Comorbidity Index. These variables statistically significantly predicted NT-proBNP, *F*(2,115) = 13.665, p < 0.001, r = 0.438. Only fluid overload added statistically significantly to the prediction with a p < 0.001 (p = 0.059 for CCI).

### Association between fluid overload, inflammatory, and cardiovascular biomarkers

Laboratory parameters of inflammation (CRP and SAA) were not significantly elevated in the fluid overloaded patient group (Table 2). Likewise, we did not observe a statistically significant association in correlation analyses between these parameters and fluid overload (r = 0.108/p = 0.253 for CRP, r = 0.124/p = 0.277 for serum amyloid A). Furthermore, no association was detected between interdialytic weight gain and the mentioned markers (data not shown).

**Figure 3:**
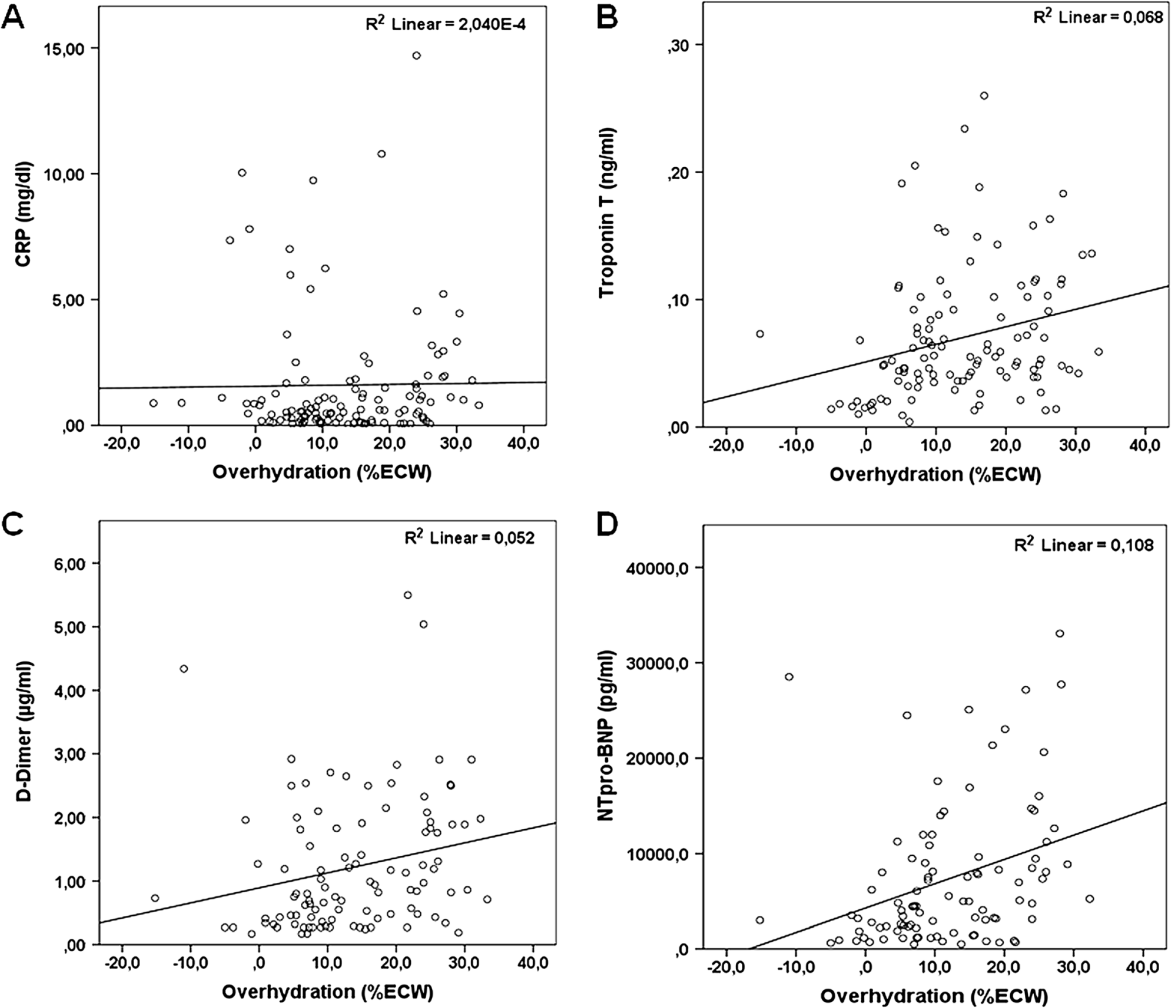
**Relationship between fluid overload and markers representing inflammatory processes and cardiac strain markers. (A)** C-reactive protein (cases >15 mg/dl excluded, n = 1), **(B)** D-Dimer (cases >7.5 μg/ml excluded, n = 3), **(C)** troponin T (cases >0.45 ng/ml excluded, n = 4) and **(D)** NT-proBNP (cases >35,000 pg/ml excluded due to assay incapacity, n = 26).

However, parameters of coagulation (D-Dimer), myocardial ischemia and cardiac strain (troponin T and NT-proBNP) were significantly higher in the fluid overloaded group (Table 2). Patients below the median NT-proBNP value of 7536 pg/ml showed significantly lower percental fluid overload (mean 9.5 ± 8.7%) compared to those above (17.7 ± 9.3%; Additional file 2: Figure S2). We further observed a significant positive association between fluid overload and D-Dimer (r = 0.316/p = 0.001), troponin T (r = 0.325/p < 0.001) and NT-proBNP (r = 0.405/p < 0.001; Figure 3). Of note, blood pressure measurements before hemodialysis and the mean number of antihypertensive drugs per patient were similar between normohydrated and fluid overloaded patients (Table 2).

In a multivariate regression analysis, troponin T showed to be independently associated with fluid overload (standardized coefficient beta 0.237/p = 0.007), with a strong confounder found in age (beta 0.372/p < 0.001), while no influence could be shown for logNT-proBNP (beta -0.006/p = 0.958) or NT-proBNP groups below or above 35,000 pg/ml (beta 0.117/p = 0.256). Documented coronary artery disease was also not associated with higher troponin T levels in this model.

## Discussion

In the present analysis, we evaluated volume status in a chronic hemodialysis cohort of 244 patients from 3 hemodialysis centers. First, we observed that a high percentage of patients (39%) exhibited considerable pre-HD fluid overload. Secondly, we were able to confirm an inverse association between the degree of fluid overload in hemodialysis patients and their body mass index as well as serum albumin. Third, an association was shown for fluid overload and biomarkers of cardiovascular compromise, but not with interdialytic weight gain or blood pressure - which stands in contrast to a study by Passauer et al. where a weak positive correlation between pre- and post-dialytic systolic blood pressures and fluid overload was shown in non-diabetic subjects on hemodialysis [22].

The percentage of patients who met the definition criteria of fluid overload surpassed the previously estimated rate of 20% in European HD centers [2]. The highest rate of fluid overload was detected in the University-based dialysis unit, possibly because patients from this center comprised a collective with more comorbidities and were therefore more prone to chronic fluid overload. This state of potentially higher morbidity was also associated with significantly lower average body mass in patients dialyzing at the University-based hemodialysis unit.

Lower body mass is known to be strongly associated with increased mortality in the hemodialysis population in general [32] and in fluid overloaded dialysis patients in particular [33]. The same applies for yet another patient cohort: those suffering from congestive heart failure (CHF). For the CHF population, the association between low body mass index and mortality has been shown in the CHARM trial in 2007 as well as other studies analyzing mortality in this population [34,35].

An additional association from our study supporting these findings is the inverse relationship between increasing fluid overload and nutritional status represented by serum albumin and total protein levels. Several factors - patients’ comorbid conditions, malnourishment, a certain dilution effect - might be responsible for hypoalbuminemia. This finding has been described previously in a non-dialysis-dependent chronic kidney disease patient group [36] as well as a chronic hemodialysis cohort [2]. In summary, underweight and malnourished, but not necessarily older or more obese patients tend to show higher degrees of fluid overload.

A recent study in 79 dialysis patients aiming at a reduction of blood pressure and antihypertensive medication, yielded interesting results with regard to a lower percentage of body fat in fluid overloaded dialysis patients [37]. These findings are in accordance with our results enforcing the view that underweight patients are more susceptible to fluid overload, whereas adipose patients tend to be in an underhydrated condition.

The so-called reverse epidemiology in the hemodialysis population has been a ‘hot topic’ of the past years and it has been postulated that inverse relationships between mortality and classical cardiovascular risk factors, i.e. body mass index and hypertension, may exist in hemodialysis patients [33,38]. Our results further lend support to this concept, as we could demonstrate that lean patients are generally more fluid overloaded while it is known that fluid overloaded individuals comprise the more morbid patient group with higher markers of the malnutrition-inflammation complex syndrome (MICS) [18,39].

This observation might be explained by clinical misclassification - patients appear fluid overloaded due to their physique, when they are in fact normo- or even dehydrated - and/or deliberately insist on dry weight reduction despite normohydration. Clearly, further studies are necessary to delineate the underlying mechanisms of these observations.

The other important finding in our analysis was the statistically significant association of fluid overload and biomarkers representing cardiovascular damage and strain, notably TnT and NT-proBNP. These results correlate well to those by Velasco et al., who used a value similar to fluid overload (time-averaged fluid overload, TAFO) for 30 patients who underwent bioimpedance assessments at three consecutive dialysis sessions. Additionally, these patients underwent cardiac MR imaging in order to quantify left ventricular hypertrophy. The clear association of higher TAFO with higher left ventricular mass index confirms previous assumptions but, as is mentioned in their study limitations section, patients might have represented a study population ‘above the mean’, as they underwent HDF with few hypotensive episodes; additionally, the measured BNP levels were rather low compared to a previous study by Sommerer et al. [14,40]. In our multivariate analysis, TnT proved to be independently associated with fluid overload, but no correlation was observed between TnT and NT-proBNP as well as documented coronary artery disease, which points toward cardiac damage potentially being caused directly by fluid overload and questions the applicability of TnT in the diagnosis of coronary events in hemodialysis patients.

Interestingly, no difference was shown for pre-HD blood pressure measurements between normohydrated and fluid overloaded patients; a fact that could foster a budding theory that the elevation of NT-proBNP and the generation of cardiac insufficiency are rather caused by constant volume overload than hypertension-induced damage in hemodialysis patients. It has previously been shown that, regardless of fluid overload, blood pressure values vary widely. As other factors besides fluid overload, such as sympathetic nervous activity, the renin-angiotensin system, cardiac function and potentially the interdialytic weight gain, also contribute to the genesis of arterial hypertension in hemodialysis patients, it appears challenging to associate the volume status with blood pressure [41,42].

Furthermore, we could show that fluid overload is also associated with increased D-Dimer levels, a parameter representing thrombotic events and a state of coagulatory activation. It is of note that D-Dimer has previously been shown to be particularly elevated in hemodialysis patients with a central venous catheter compared to arteriovenous fistulas [43]; this was confirmed by our analysis (data not shown). As no statistically significant difference was observed regarding dialysis access in normohydrated versus fluid overloaded patients, the observed difference of D-Dimer levels between the two groups might probably be due to another cause, which has yet to be determined.

We acknowledge the following limitations to our analysis: The presented data are descriptive; in the context of the recently performed ‘BVM-Reg’ study further data will become available and contribute to our current knowledge concerning fluid overload, blood-volume monitoring-guided dry weight reduction and cardiovascular parameters. Further, for many patients psychological issues interrelated with their physique and dry weight might lead to clinical misclassifications regarding their ideal weight as cachectic patients do not wish to further reduce it whereas obese patients might insist on further lowering it. Also, no study-associated cardiac imaging or functional testing was carried out, measures which could potentially be of interest for the exclusion of structural heart disease. It therefore cannot be clearly determined whether NT-proBNP values are solely raised due to fluid overload and renal secretion incapacity or if additional cardiac pathologies, i.e. congestive heart failure, contribute in these patients. NT-proBNP has been described to be of significant value as a marker both of fluid overload [11] as well as of left ventricular dysfunction [4]; both factors seem to show a significant overlap in this special population [44].

## Conclusions

In conclusion, our data show that a significant percentage of middle European maintenance hemodialysis patients are chronically fluid overloaded. Fluid overload is most common in patients with low body mass index and lower serum albumin levels, in accordance with the previously described reverse epidemiology in hemodialysis patients. Additionally, fluid overload is associated with higher levels of biomarkers representing activated coagulation and cardiac muscle decomposition and ischemia, supporting the hypothesis that fluid overload plays a significant role in the generation and augmentation of vascular and cardiac damage. Fluid overload has emerged as a parameter that strongly correlates with cardiovascular biomarkers but seems to be independent of inflammation as well as elevated blood pressure in hemodialysis patients. Therefore, we propose that fluid overload could be defined as an independent single entity - equivalent to a biomarker - with the potential to be introduced for intervention guidance.

## Competing interests

The authors declare no competing financial interests.

## Authors’ contributions

MA and MHe were responsible for the study conception, patient recruitment, analysis and interpretation of data and manuscript drafting and editing. MHa, JW and JK contributed to patient recruitment and manuscript editing. GP and ME carried out patient recruitment, BCM measurements and data acquisition at centers 2 and 3. DB and WH were involved in the process of manuscript drafting, scientific discussion of the manuscript and critical revision. MS was responsible for study design and supervision, manuscript drafting and editing. All authors read and approved the final manuscript.

## Pre-publication history

The pre-publication history for this paper can be accessed here:

http://www.biomedcentral.com/1471-2369/14/266/prepub

## Supplementary Material

Additional file 1: Figure S1 Linear regression analysis of body mass index and fluid overload. Click here for file

Additional file 2: Figure S2 Low versus high NT-proBNP groups were assessed for percental fluid overload. The median for NT-proBNP was calculated and patients below (n = 62) and above the median (n = 61) were compared with Student’s t-Test. Click here for file
