# Supplementary figures and images for: Fluid overload in hemodialysis patients: a cross-sectional study to determine its association with cardiac biomarkers and nutritional status
Source: BMC Nephrol. 2013 Dec 2;14:266. doi: 10.1186/1471-2369-14-266 (PMC4219439; doi:10.1186/1471-2369-14-266)

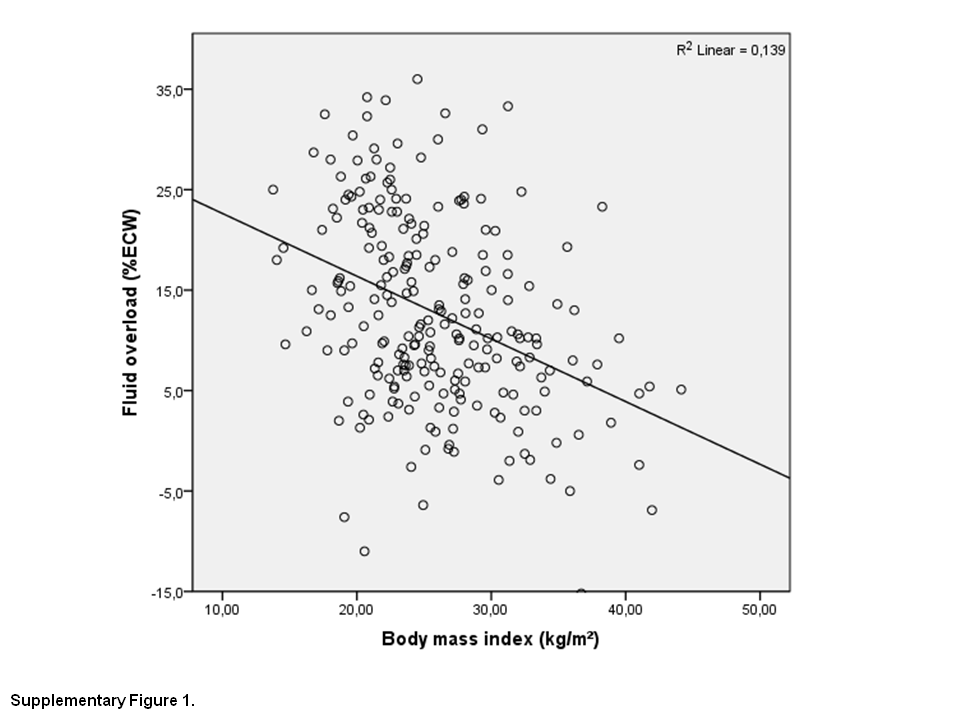

Supplement: Additional file 1: Figure S1 — Linear regression analysis of body mass index and fluid overload. [file 1471-2369-14-266-S1.tiff]

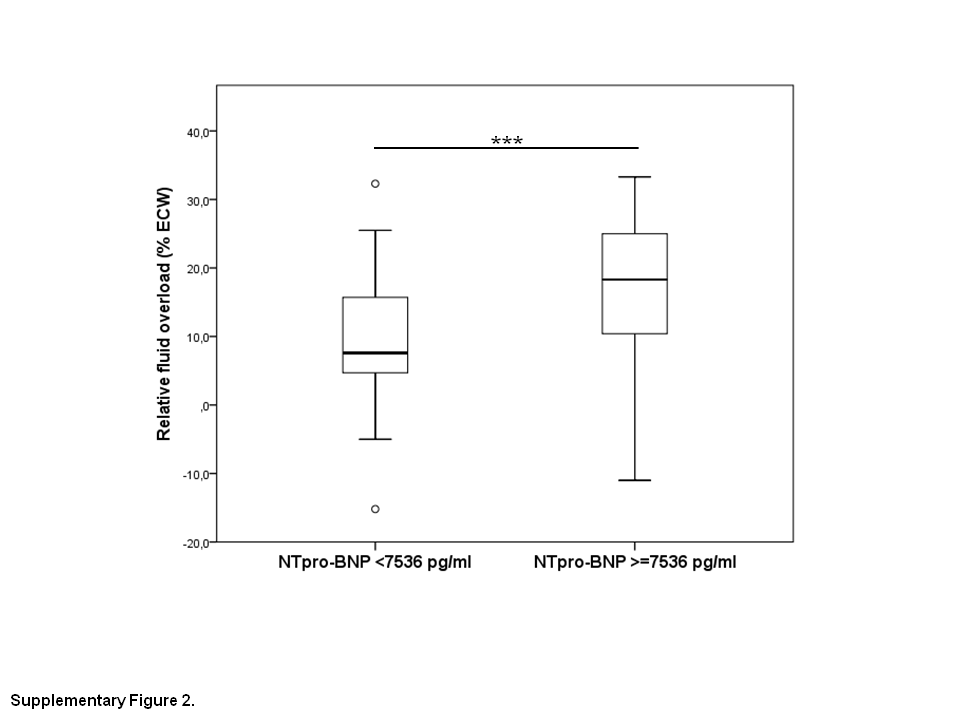

Supplement: Additional file 2: Figure S2 — Low versus high NT-proBNP groups were assessed for percental fluid overload. The median for NT-proBNP was calculated and patients below (n = 62) and above the median (n = 61) were compared with Student’s t-Test. [file 1471-2369-14-266-S2.tiff]
